# Barriers and opportunities for nontraditional social work during COVID-19: Reflections from a small LGBTQ+ nonprofit in Detroit

**DOI:** 10.1177/1473325020984156

**Published:** 2021-03

**Authors:** Angela K Perone

**Affiliations:** School of Social Work, University of Michigan, Ann Arbor, MI, USA

**Keywords:** Advocacy, ageing, community work, LGBTQ, professionalism

## Abstract

COVID-19 has significantly changed individual lives and organizational structures throughout the world. Certain regions and populations, however, have experienced the effects of this global pandemic to a heightened degree. This article includes reflections from a small LGBTQ+ nonprofit in Detroit, a city with some of the starkest health and economic disparities in the United States. While COVID-19 has illuminated numerous barriers for LGBTQ+ older adults in Detroit, it has also revealed surprising ways that nontraditional social work is emerging. The experiences from this organization suggest that when faced with limited resources, LGBTQ+ community members and advocates have expanded their services in ways that mirror the work of many professional social workers, including interpersonal support; facilitated group discussions; direct services; program design, delivery, and coordination; community organizing; and research. Through these roles, community members are beginning to serve as nontraditional social workers to address the urgent and unmet needs of LGBTQ+ older adults and increase the visibility of this population during COVID-19.

COVID-19 has significantly changed individual lives and organizational structures throughout the world. Certain regions and populations, however, have experienced the effects of this global pandemic to a heightened degree. I write this reflection to capture the experiences I have seen while pursuing a doctoral degree in social work and serving as Executive Director of a small nonprofit organization that serves lesbian, gay, bisexual, transgender, queer, and same-gender-loving (LGBTQ+) older adults in Detroit, Michigan during COVID-19. This nonprofit formed five years ago after existing informally as a coalition of community leaders, LGBTQ+ older adults, and service providers who recognized a gap in services to this community.

Detroit, Michigan is a city that reflects some of the starkest disparities in the United States. Decades of racial and residential segregation and white flight from the city have produced a city with one of the largest concentrations of Black Americans in any major city in the United States (82.7%) (United States Census, 2020) and a significant population of Black Americans who identify as LGBTQ+ and/or who are living with HIV. While Detroit’s overall population has declined, its aging population continues to grow. Detroit is one of the poorest cities in the United States where residents have some of the highest number of health conditions (Bach, 2020). Some residents live in zip codes in Detroit with life expectancies lower than North Korea (MacDonald & Ramirez, 2016). Not surprisingly, Detroit has one of the highest proportions of COVID-19-related deaths among United States cities.

## Invisibility at multiple levels

We refuse to be invisible. That is the tagline of our organization. We serve LGBTQ+ older adults in Detroit and the surrounding areas in Southeast Michigan. However, COVID-19 has underscored myriad ways that this population remains invisible, and in some ways, COVID-19 has exacerbated this invisibility.

Many LGBTQ+ older adults that we serve rely on other LGBTQ+ older adult friends (“chosen family”) for caregiving needs (e.g. transportation to doctor’s appointments or grocery shopping, meal preparation, face-to-face emotional support). COVID-19 has fractured these support networks, given stay at home orders and concern about transmission within this population. For example, a monthly dinner at a local community center provided discussions and emotional support for LGBTQ+ older Black men. Some of the men in this group have HIV. After losing dear friends to AIDS, this in-person group provided a new network of support. When this community center closed due to COVID-19, this group had no place to meet. Few of the participants had tablets or computers to maintain a virtual group. When they were able to regularly meet at a community center for older adults, their needs and experiences were visible. They received tangible support from each other as well as the community center providing the support. However, when the community center moved most of its programming to a virtual platform, most of these older adults were left without tools to connect. Their needs and experiences were essentially rendered invisible and the group ceased to exist.

The digital divide has also left many LGBTQ+ older adults in Detroit without much-needed information. Resources are constantly evolving during this pandemic. Organizational needs and services rapidly change as funding becomes more or less available. Most organizations provide updated information about their services through their website. However, LGBTQ+ older adults who lack tablets, computers, or Internet lack access to this information. Few organizations have been able to provide resources to tackle the digital divide. Without providing these resources, LGBTQ+ older adults without computers, tablets, and/or access to the Internet remain invisible to many community organizations providing services during COVID-19.

Even among LGBTQ+ older adults who do have access to this technology, some may still be reticent to reach out for help. Decades of discrimination from government officials and service providers have made many weary of seeking help outside a close-knit caregiving support network. But, fractured support networks during COVID-19 have necessitated an expansion of support. For many LGBTQ+ older adults who have experienced multiple types of discrimination together (e.g. discrimination based on race, gender, and sexual orientation), access to culturally responsive service providers is critically important. One woman reached out to our organization for help with food only after she had resorted to eating pet food. She was terrified to seek help from general aging service providers because she was fearful that they would deem her unable to care for herself and compel her to move to a nursing facility where her risk of COVID-19 would increase. She had come of age during a time when being gay meant you could be fired, jailed, or potentially lobotomized. She also recounted numerous instances of discrimination and harassment by strangers, employers, and landlords throughout her life. While her feared scenario is highly unlikely given her situation, her previous experiences of discrimination made her particularly hesitant to seek help. Only after she had completely run out of food did she turn to anyone for help—and she only sought help from an LGBTQ+ organization focused on serving older adults. The digital divide did not keep her invisible from service organizations. However, her past experiences of discrimination left her invisible to organizations until she had no other choice to make herself—and her needs—known.

LGBTQ+ older adults in Detroit also remain invisible in COVID-19 data collection efforts. Most states in the United States collect data by race, including Michigan. However, only two states (Pennsylvania and California) also collect data on sexual orientation and gender identity. Michigan does not currently collect this data. The experiences of LGBTQ+ older adults, including LGBTQ+ older adults of color, thus, are not being documented. Data collection is vital for ensuring equal access to services and to better understand health disparities. Research outside of COVID-19 has already revealed that LGBTQ+ older adults experience higher rates of health disparities compared to heterosexual, cisgender older adults (Fredriksen-Goldsen et al., 2013, 2014). COVID-19 may exacerbate these disparities. However, without data on sexual orientation and gender identity, the information gathered remains incomplete and LGBTQ+ older adults remain invisible when researchers and policymakers review and interpret this data.

As a doctoral student at a top social work school, I have access to significant resources regarding research (e.g. libraries, journal subscriptions, methodology experts). This role places me at significant privilege to see the power of research and the capital and resources that can flow from it. I have access to a scholarly world that many of the people I serve do not. In contrast, the community and organization I serve lack much of this privilege in terms of financial resources and social and cultural capital. Health disparities during COVID-19 are not limited to individual differences but to decades of historical and systemic oppression. Straddling these worlds during COVID-19 has only heightened my desire to help build a body of research that addresses the needs of LGBTQ+ older adults. Data collection on sexual orientation and gender identity is but one example of how research can shape the resources – and lives – of a community. COVID-19 has also underscored the need for research that makes visible the myriad inequitable structures and processes (both in research and beyond) that render many LGBTQ+ older adults invisible and often unserved.

## Caregiving and community support

Despite the barriers that COVID-19 has presented–and that have increased during COVID-19 for LGBTQ+ older adults—I have been heartened by the ways in which LGBTQ+ community has creatively navigated these barriers to identify new solutions. One of the first and most immediate needs we encountered was access to food. Some older adults that reached out to our organization relied on governmental programs that had stringent requirements on how they could access food. For example, one older adult had sufficient funds on a government issued card to purchase food, but his multiple health conditions made grocery shopping unsafe. His caregiving network included older adults who were in a similar situation. Prior to COVID-19, our organization did not provide direct services outside our Friendly Caller Program, a telephone buddy program that matches volunteer callers with LGBTQ+ older adults to build connections and reduce social isolation. Our program manager, however, was determined to get him food. She made countless calls to other service providers to identify resources for him. Few surfaced that could provide food delivery to him. Initially, her calls revealed that government restrictions also precluded someone from purchasing food for him with his card. However, her persistent advocacy ultimately led to information about a cumbersome process for appointing someone who could use his card to purchase food. While that remains a possible long-term solution during COVID-19, it does not provide immediate access to food. She then worked closely with other LGBTQ+ organizations to identify volunteers who could purchase and deliver groceries to him to meet his needs now. We then collectively crafted a narrative to funders to demonstrate need for expanded programming that includes funding for food. During this time, other LGBTQ+ older adults contacted us for food assistance. Our program team of part-time workers and volunteers then reached out to their own networks in the LGBTQ+ community to identify additional volunteers who could purchase and deliver food to older adults.

Volunteers have found creative ways to help and have facilitated new connections within the LGBTQ+ community. Prior to COVID-19, most of our volunteers were older adults. Now, many of our volunteers are under 35. One such volunteer match underscores how new caregiving relationships have blossomed amidst COVID-19. Each week, one of our youngest volunteers delivered food to her LGBTQ+ older adult recipient. The pair also engaged in conversation (while maintaining physical distance and wearing masks and/or face shields). Even though masks and face shields kept various parts of the volunteer’s face invisible, she found creative ways to emote and build rapport using her hands, arms, and body. After several visits, she started bringing one single flower to her recipient to make her feel special. When so many other structures and processes had rendered her invisible, these simple gestures provided much needed compassion and dignity that moved beyond basic needs.

Volunteers and part-time program coordinators also developed new programming that addresses multiple barriers at once through virtual multigenerational dinners and discussions. We secured funds to deliver tablets and provide internet access to LGBTQ+ older adults through a pilot program in Detroit. We also held virtual tech workshops to help participants understand how to use their tablet, access the Internet, and use Zoom, the digital platform used for the dinner and discussion. We coordinated with a food delivery service to ensure that each participant had a dinner from a restaurant of their choice (up to a certain dollar value) in their geographic region. The first dinner took place after police officers in Minneapolis killed George Floyd—a Black man suspected of using counterfeit money to buy cigarettes—during a chokehold. This event served as a catalyst for a surge of Black Lives Matters protests throughout the United States, including Detroit. During this virtual dinner, facilitators (both paid part-time program coordinators and volunteers) led small-group discussions that focused on both COVID-19, race and racism, and how these experiences intersect.

To address the needs of finding culturally-responsive LGBTQ+ welcoming services, volunteers and part-time coordinators are also creating an interactive website and hard copy guide that includes both COVID-19 resources for LGBTQ+ older adults. To curate these resources, volunteers have identified LGBTQ+ welcoming organizations and businesses through community networks and word-of-mouth. Each organization listed has signed a Statement of Nondiscrimination and completed a form indicating whether it has received a diversity, equity, and inclusion (DEI) training and an LGBTQ+-specific training in the past two years. Organizations also identified whether they had written nondiscrimination policies for workers and service recipients that include sexual orientation, and gender identity or expression.

Data collection based on sexual orientation and gender identity remains an elusive goal in Michigan. However, as other states have begun to take steps to do this (e.g. Pennsylvania, California), LGBTQ+ community organizations in Southeast Michigan, including Detroit have begun seeking assistance from research partners at local universities to design a community-based survey to track the experiences and needs of LGBTQ+ older adults during COVID-19.

## Nontraditional social work

The need for social work is strong during COVID-19, and many trained social workers are delivering critical supports for individuals across the United States. However, the urgency of this moment and immense need for services may be invoking nontraditional social work, too. The experiences at this organization illuminate how nontraditional social work has emerged.

COVID-19 has created a bevy of complex and urgent needs that our current support structures are not always designed to address, particularly in the timely manner that the needs demand. LGBTQ+ older adults, especially those with compromised immune systems, cannot simply pick up food from the grocery store when it is needed. Many organizations with food boxes require pick-up from a designated location, which creates additional barriers for this population. Updated information about COVID-19 resources are often contained on websites that lack specific information about LGBTQ+ culturally responsive services and/or that few people can reach due to a digital divide between those who have access to technology to access this information and those who do not. All of these structures have left LGBTQ+ older adults in Detroit invisible, even though they comprise a population with high need for these services.

Nevertheless, LGBTQ+ community members and advocates have stepped forward to identify creative ways to navigate these barriers and address the needs of LGBTQ+ older adults during COVID-19. Most of these individuals lack professional training or educational background as a social worker. However, in many ways, they are functioning as nontraditional social workers. Paid part-time workers, including our program manager and program coordinators have worked diligently to identify community resources for food and technology and created a network of volunteers to deliver direct services. None of them have social work training or degrees. They have also designed new programs to facilitate group discussion and provide support about experiences of COVID-19 and racial injustice. These programs operate like support groups but without facilitators with professional social work degrees to lead them. Community volunteers without social work backgrounds deliver direct services to LGBTQ+ older adults while also providing emotional support to isolated community members. Community members and part-time workers without professional community organizing experience have collaborated to create a new network of community volunteers to deliver programs and provide updated information about culturally responsive resources for LGBTQ+ older adults in the Detroit Metro region. To address insufficient data on LGBTQ+ older adults, LGBTQ+ community members and organizational leaders have come together to design a survey and collect data among LGBTQ+ older adults. Few organizational participants have a social work background; however, we have solicited assistance from social work researchers. Community participants will comprise a community research advisory committee to guide the research.

While COVID-19 has illuminated numerous barriers for LGBTQ+ older adults in Detroit, it has also revealed surprising ways that nontraditional social work is emerging. The experiences of our organization suggest that when faced with limited resources, LGBTQ+ community members and advocates have expanded their services in ways that mirror the work of many professional social workers, including interpersonal support; facilitated group discussions; direct services; program design, delivery, and coordination; community organizing; and research. Through these roles, community members are beginning to serve as nontraditional social workers to address the urgent and unmet needs of LGBTQ+ older adults and increase the visibility of this population during COVID-19.
